# An antimicrobial blue light device to manage infection at the skin-implant interface of percutaneous osseointegrated implants

**DOI:** 10.1371/journal.pone.0290347

**Published:** 2023-08-25

**Authors:** Jemi Ong, Alexa Nazarian, Joshua Tam, William Farinelli, Sandeep Korupolu, Lynn Drake, Brad Isaacson, Paul Pasquina, Dustin Williams

**Affiliations:** 1 Department of Biomedical Engineering, University of Utah, Salt Lake City, UT, United States of America; 2 Department of Orthopaedics, University of Utah, Salt Lake City, UT, United States of America; 3 Wellman Center for Photomedicine, Massachusetts General Hospital, Boston, MA, United States of America; 4 Department of Dermatology, Harvard Medical School, Boston, MA, United States of America; 5 The Geneva Foundation, Tacoma, WA, United States of America; 6 Department of Physical Medicine and Rehabilitation, The Musculoskeletal Injury Rehabilitation Research for Operational Readiness (MIRROR), Uniformed Services University, Bethesda, MD, United States of America; 7 The Center for Rehabilitation Sciences Research, Uniformed Services University, Bethesda, MD, United States of America; 8 Department of Rehabilitation, Walter Reed National Military Medical Center, Bethesda, MD, United States of America; 9 Department of Pathology, University of Utah, Salt Lake City, UT, United States of America; University of Vigo, SPAIN

## Abstract

Antimicrobial blue light (aBL) is an attractive option for managing biofilm burden at the skin-implant interface of percutaneous osseointegrated (OI) implants. However, marketed aBL devices have both structural and optical limitations that prevent them from being used in an OI implant environment. They must be handheld, preventing even irradiation of the entire skin-implant interface, and the devices do not offer sufficient optical power outputs required to kill biofilms. We present the developmental process of a unique aBL device that overcomes these limitations. Four prototypes are detailed, each being a progressive improvement from the previous iteration as we move from proof-of-concept to *in vivo* application. Design features focused on a cooling system, LED orientation, modularity, and “sheep-proofing”. The final prototype was tested in an *in vivo* OI implant sheep model, demonstrating that it was structurally and optically adequate to address biofilm burdens at the skin-implant of percutaneous OI implants. The device made it possible to test aBL in the unique OI implant environment and compare its efficacy to clinical antibiotics–data which had not before been achievable. It has provided insight into whether or not continued pursual of light therapy research for OI implants, and other percutaneous devices, is worthwhile. However, the device has drawbacks concerning the cooling system, complexity, and size if it is to be translated to human clinical trials. Overall, we successfully developed a device to test aBL therapy for patients with OI implants and helped progress understanding in the field of infection management strategies.

## 1 Introduction

Osseointegration (OI) involves the direct skeletal attachment of a prosthesis to individuals with limb loss and has become increasing popular for individuals across the globe with transfemoral amputation. Superficial infections affect up to 50% of patients with transfemoral percutaneous osseointegrated implants [1]. Although bacterial contamination at the implant site is unavoidable, antimicrobial blue light (aBL) may act as a preemptive measure against infection [2]. Utilized as a routine treatment, irradiating the skin-implant interface with aBL may inhibit bacterial load from ever exceeding the level of infection (~10^5^ CFU) [3, 4]. However, before the device presented in this paper was developed, no existing aBL system was capable of testing this concept for OI implant applications; we present an iterative process of a unique aBL device for application at the skin-implant interface of percutaneous OI implants. While the design and prototypes are specific to an OI implant sheep model, the long-term goal is to optimize the device for human clinical trials and application.

Current aBL devices are limited both structurally and optically. Marketed aBL units are often built with small light emitting diode (LED) arrays or pen-like contraptions with optical fibers (Luminance RED Acne Device). Some companies have tried attaching LEDs to the inside of full-face masks (Omnilux and Cleopatra masks). However, none of these existing designs can be utilized in transfemoral OI implant systems. The arrays and pen devices are handheld, which would necessitate constant device reorientation to ensure even irradiation of the circumferential skin-implant interface. Not only would this be inconvenient, but a patient would likely miss a section(s), leaving bacteria free to proliferate. The aforementioned face mask is unique in that it is strapped onto a patient’s head, which guarantees that the entire face is evenly irradiated and allows a patient to go about normal activities during treatment. Yet, despite this innovative design, the aBL face mask cannot be directly used at the skin-implant interface of OI implants.

Additionally, marketed aBL devices have low optical power outputs, which are insufficient to kill bacteria present around OI implants. The skin-implant interface is often inhabited by biofilms from the surrounding environment, or planktonic bacteria that has preferentially transitioned to the biofilm phenotype on metal implants [5, 6]. While *in vitro* studies reveal that aBL is effective against both planktonic and biofilm bacteria, much higher fluences (optical power output) are required for biofilms [7–9]. These higher fluences necessitate a cooling system to prevent overheating and bacterial pasteurization. While manufacturer websites do not specify optical power output parameters, it is evident that marketed aBL devices lack cooling systems, which limits them to lower fluences. Thus, independent of structural issues, available aBL systems cannot be utilized by patients with transfemoral amputations to manage infection as they lack the requisite power to kill biofilms.

With the goal of utilizing the device in an OI implant sheep model, the developmental process for the aBL device herein required three key design aspects. First, the aBL device needed to “mate” with the current design of an OI implant system in sheep. Second, the aBL device needed to evenly irradiate the skin-implant interface while allowing animals to move freely and perform daily activities unhindered. Third, the device had to sustain high optical power outputs capable of killing biofilms without overheating. We present the iterative process of four separate prototype devices, from proof-of-concept to a working *in vivo* unit.

## 2 Methods

### 2.1: Device 1.0

**Fig 1:**
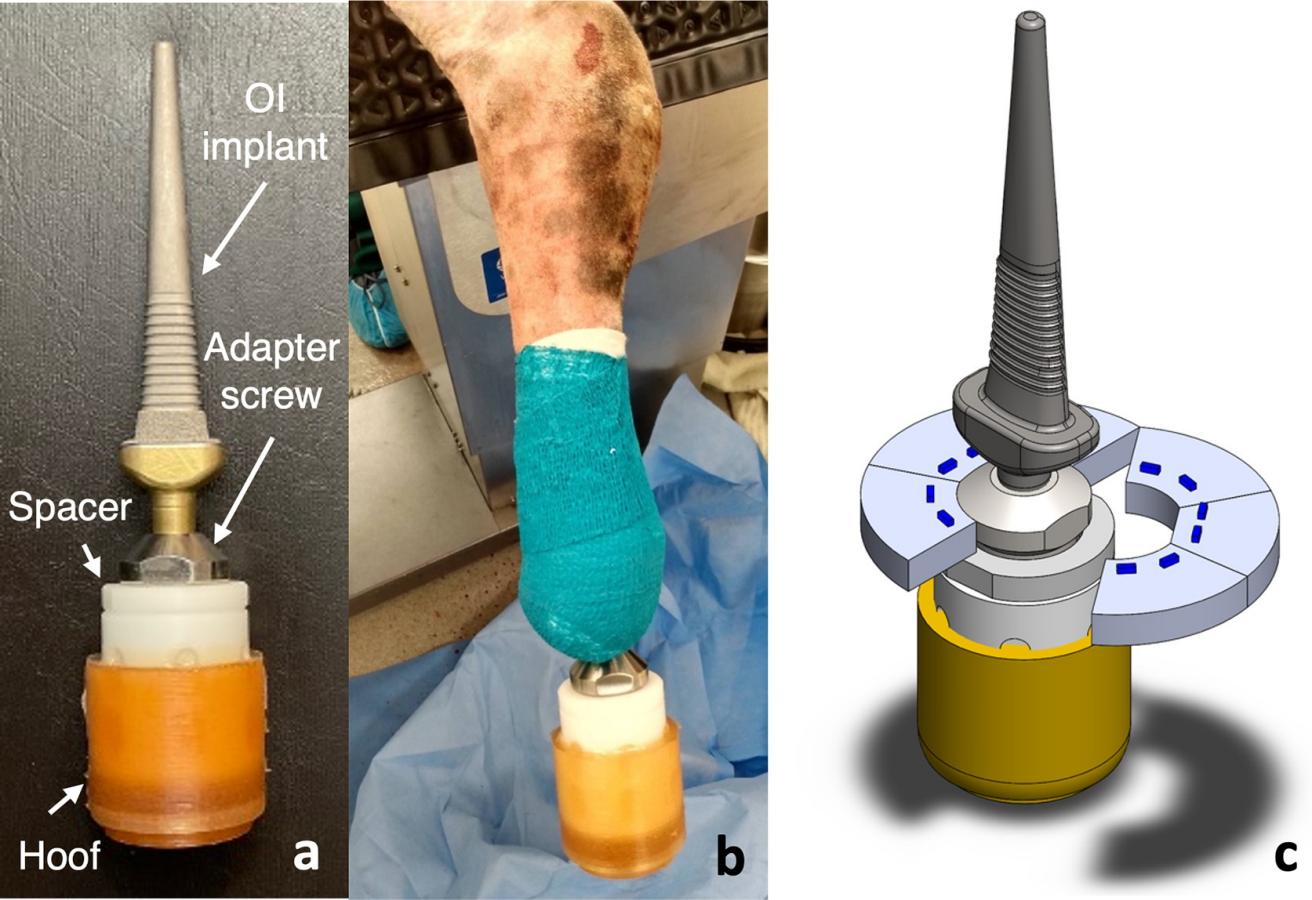
a) Assembled OI implant with the prosthetic hoof; b) example of a sheep forelimb with a surgically inserted OI implant and prosthetic hoof (from a separate IACUC-approved study); and c) proposed aBL device prototype containing two semi-circular halves situated around the adapter screw. The blue dashes represent LEDs.

The OI implant system with which the aBL device needed to “mate”, consisted of an implant, adapter screw, spacer, and prosthetic hoof (Fig 1A and 1B). This OI implant system in a sheep has been well-established and tested multiple times throughout the past years. The sheep can walk as normal, osseointegration successfully occurs, and the animals do not experience pain outside that which is to be expected from an amputation surgery [10–12]. The objective of Device 1.0 was to examine the feasibility of attaching a light emitting device comfortably and stably around a prosthetic hoof. A two-part prototype was designed: two semi-circular halves were securely situated around the prosthetic hoof’s adapter screw, allowing easy attachment and detachment without undocking the hoof system from the implant (Fig 1C).

**Fig 2:**
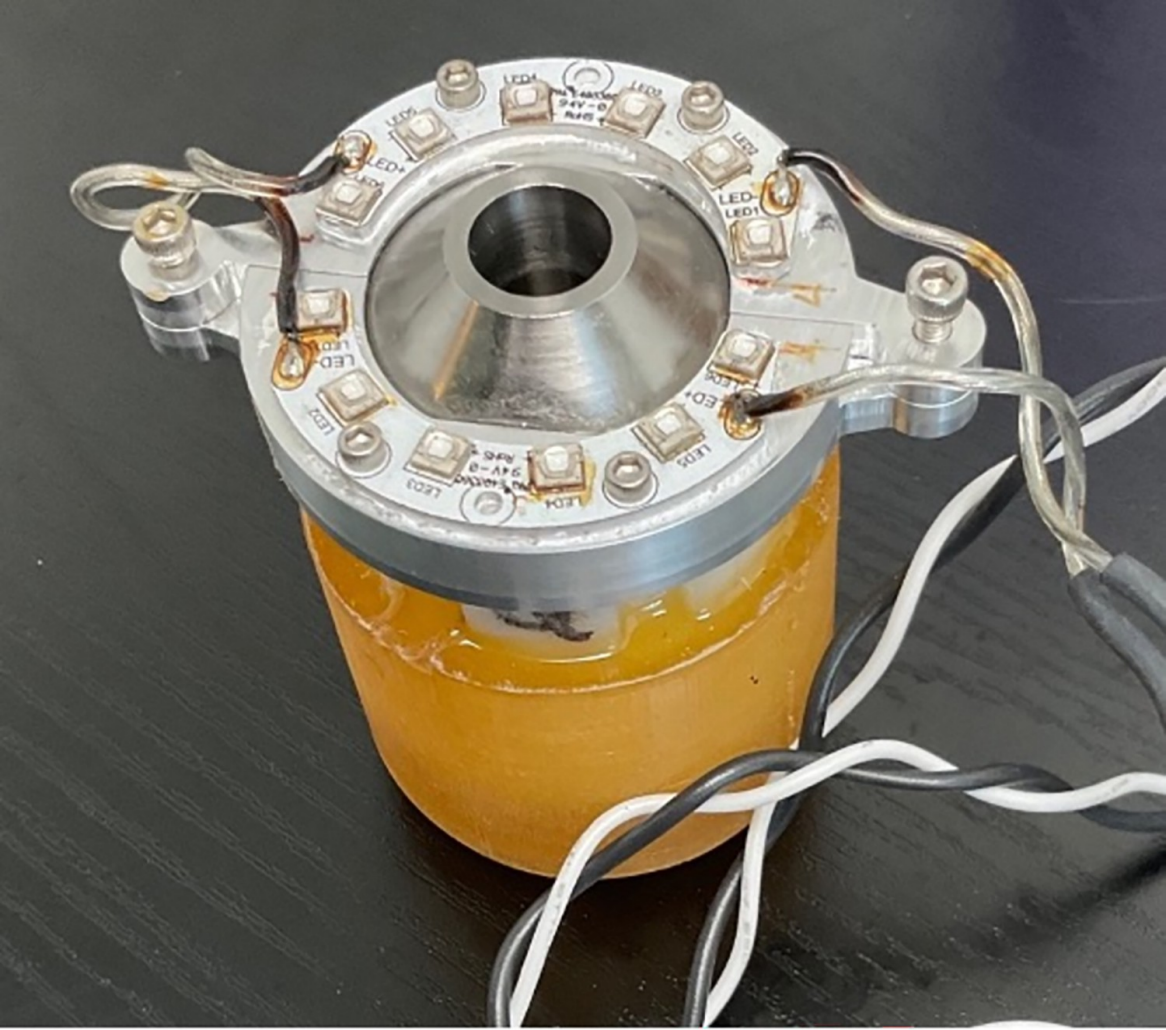
Device 1.0–12 LEDs were soldered onto two semi-circular PCBs and mounted onto an aluminum base. Two bases were connected via small screws which fit securely around the adapter screw.

Twelve 405 nm LEDs were acquired from Digi-Key Electronics supplier (model A130). LEDs were spaced 3 mm apart and soldered to two aluminum printed circuit boards (PCBs) to form a circular array (Fig 2). Each PCB was mounted onto an aluminum base, offsetting the LEDs about 5 mm from the perimeter of the adapter screw. LEDs were carefully spaced to obtain even optical power output all around the array while minimizing heat accumulation. The device was powered by a standard 25 W constant current LED driver, resulting in a 250-mW/cm^2^ optical power output when measured 1 cm above the LEDs. Output was measured using a ThorLabs power meter (Dachau, Germany).

Initial tests of the aBL unit’s functionality revealed that Device 1.0 generated excessive heat. We therefore established a heat curve for Device 1.0 against which we could compare the cooling capabilities of subsequent prototypes. A thermocouple was connected to the aluminum base and recorded the device’s temperature every five minutes for 60 min after being turned on. The test was performed three times after which results were averaged and graphed to create the heat curve.

### 2.2: Device 2.0

**Fig 3:**
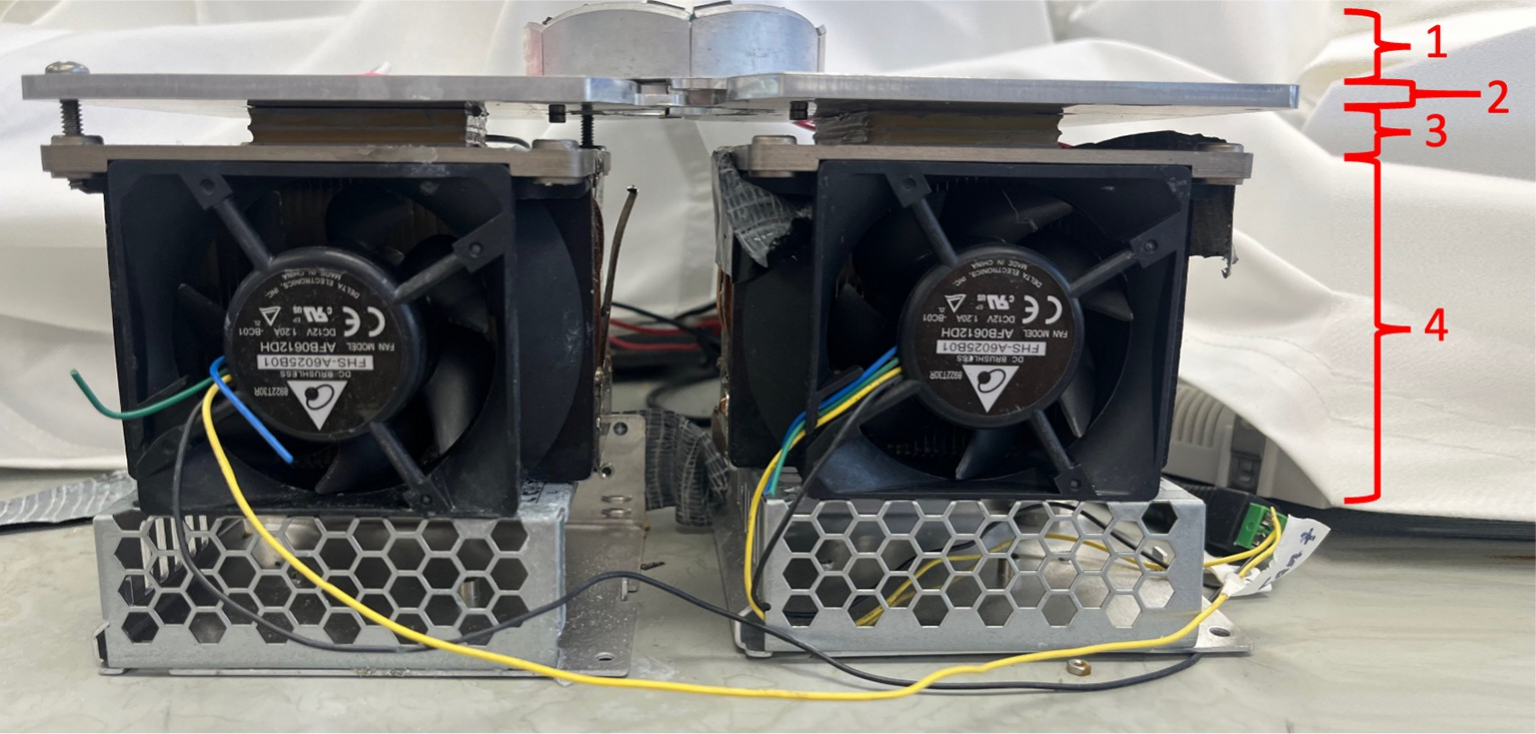
1) LED array, 2) aluminum plate bridges, 3) Peltier modules, 4) fan-cooled heat sinks.

An active cooling system was incorporated into Device 2.0 to address heat limitations of the previous prototype. It was composed of two Peltier modules with fan-cooled heat sinks (Delta Electronics; Taipei, Taiwan). These heat sinks absorbed and dissipated heat from the LEDs in addition to naturally generated heat from the Peltier modules (Joule heating). Peltier modules (CUI Devices; Tualatin, OR) were electrically driven by a temperature controller (Meerstetter Engineering; Rubigen, Switzerland) to create a cooling system that maintained the surface temperature of the skin 37°C or less. Additionally, an aluminum plate, chosen for its low thermal resistance and excellent ability to conduct heat, connected each of the two LED PCB halves to a Peltier module. The plates (manufactured by Xometry; Gaithersburg, MD) fit around the base of the LED PCBs and extended sideways to provide a heat conductive bridge between the LEDs and Peltier modules which were 40 x 40 mm each (Fig 3). Lastly, to improve heat transfer between elements, thermal paste was obtained from Omega engineering (Norwalk, CT) and applied between all interfaces.

**Fig 4:**
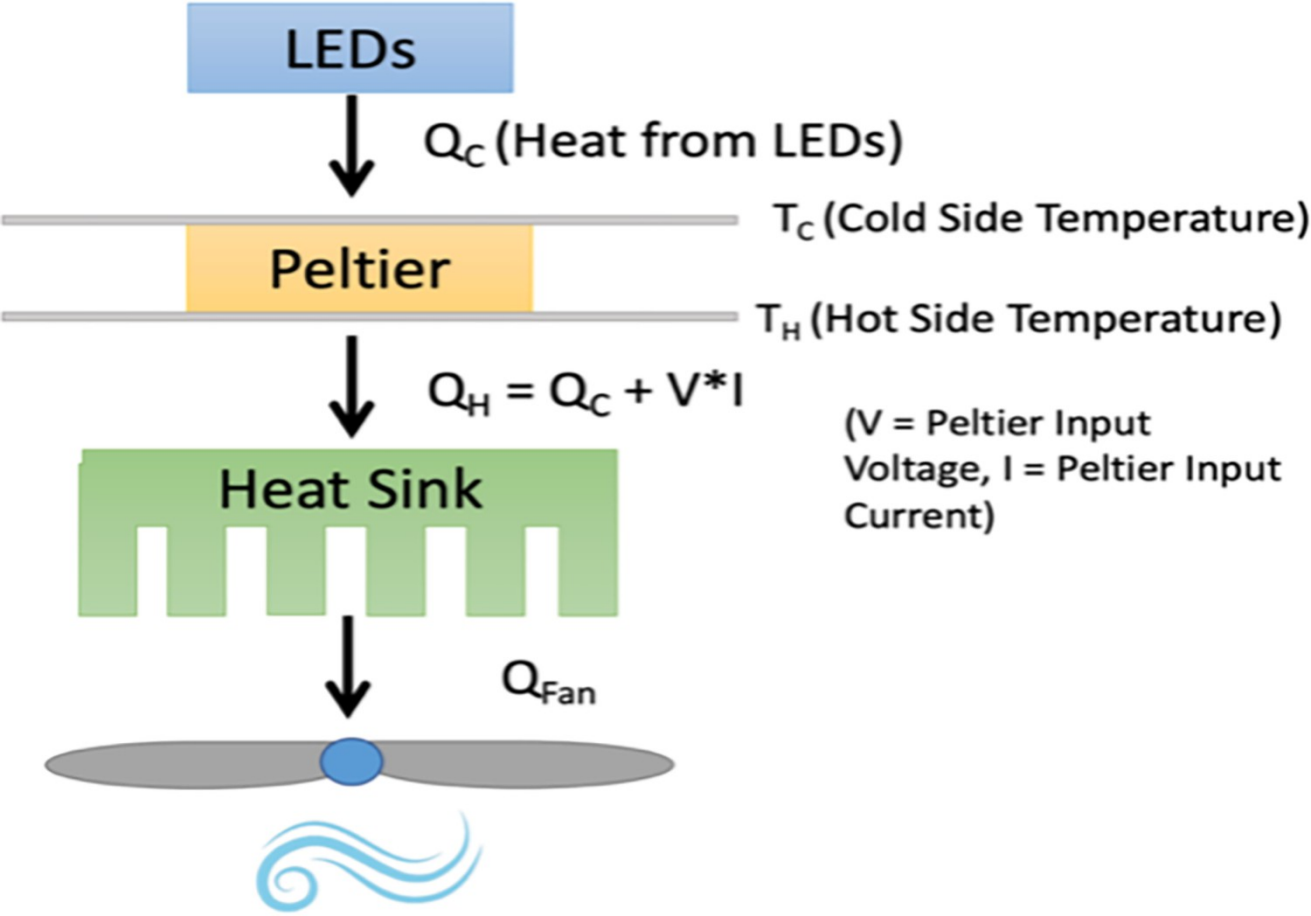
Simplified thermal schematic of active cooling system.

To prevent the LEDs from overheating, specifications for the cooling system were determined by calculating parameters based on a simple thermal schematic shown in Fig 4. The LEDs, aluminum plates, and thermal interface material were each assigned thermal resistance values. The temperature at different positions in the system were determined by Fourier’s law of heat conduction through a plane wall. The equation was re-arranged in terms of thermal resistance (R_t_) as follows:

$$\begin{array}{c} \dot{Q}=kA\frac{\left(T_{\mathrm{in}}-T_{\mathrm{out}}\right)}{L}=\frac{\left(T_{\mathrm{in}}-T_{\mathrm{out}}\right)}{R_{t}},\;\mathrm{where}\\ \dot{Q}:\;\mathrm{heat}\;\mathrm{flux}\;\mathrm{through}\;\mathrm{the}\;\mathrm{plane}\;[W]\\ k:\;\mathrm{material}’s\;\mathrm{conductivity}\;[\mathrm{W/mK}]\\ L:\;\mathrm{plane}\;\mathrm{thickness}\;[m]\\ A:\;\mathrm{plane}\;\mathrm{area}\;[m^{2}]\\ R_{t}=material’s\;thermal\;resistance\;[K/W]. \end{array}$$

It was estimated that if each LED generated about 4.8 W, an 18° C surface beneath the LEDs (T_c_) would prevent the LED junction temperature from exceeding 25°C–the data sheet’s recommended limit to ensure proper performance and lifetime of the LEDs. Maximum values and a lower than typical LED luminous efficacy (20%) were assumed to estimate the power dissipation from each LED. This ensured the development of a robust system with a heat dissipation capacity well above actual device heat generation. It was calculated that the Peltier element needed to maintain a T_c_ of about 18°C while transferring heat from the LEDs (Q_h_) in ambient conditions. To achieve the desired T_c_ and minimize joule heating, various Peltier modules were evaluated according to their Coefficient of Performance (COP; ratio of heat absorbed by the cold side (Q_c_) to power input) and heat sinks evaluated according to their thermal resistance specifications. Lower thermal resistance correlated to better heat dissipation properties and improved system efficiency. Overall, forced convection models typically provide lower thermal resistance values than natural ones, so a heat sink combined with a fan was chosen.

### 2.3: Device 3.0

**Fig 5:**
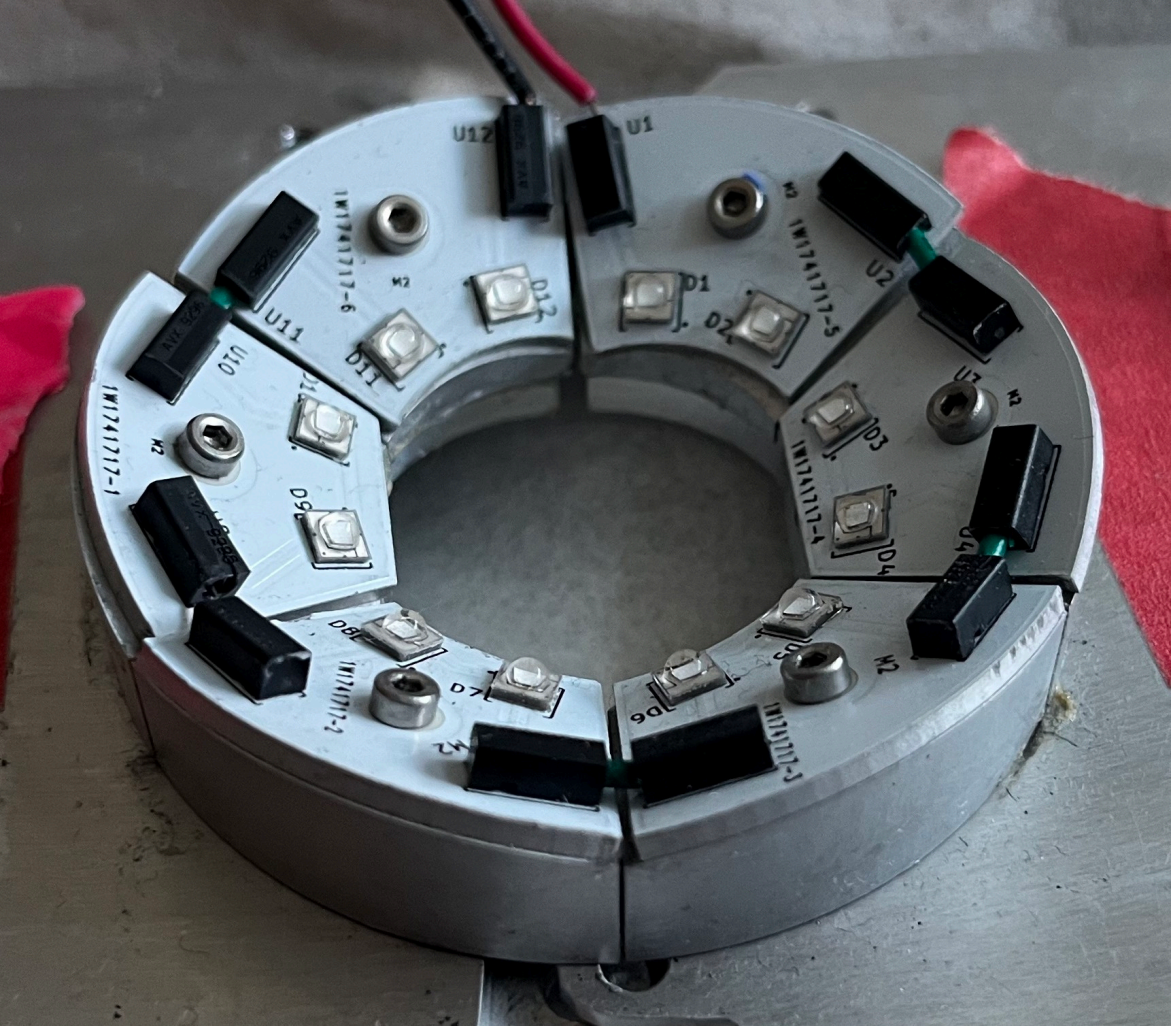
Device 3.0 with LEDs tilted 30° towards the center of the device.

With initial *ex vivo* testing on Device 2.0, there were concerns that the LEDs did not focus on the site of bacterial inoculation due to the LED’s 5 mm offset from the perimeter of the adapter screw. It was hypothesized that maximum optical power output occurred directly above the LEDs. If supported, the LEDs would need to be tilted towards the center of the array to irradiate the skin-implant interface (site of bacterial inoculation) with the highest possible optical power. To investigate the hypothesis, a tilt test was performed to measure optical power output (mW) directly above the LEDs (non-infected site) and to the side (center of the circular array; infected area). Flat and angled (30° towards the center of the device) LED orientations were tested, corresponding to Device 2.0 (Fig 2) and 3.0 (Fig 5), respectively. A power meter was clamped 1 cm above the LEDs and optical power output was recorded in 0.5 cm increments across the diameter of the device. The test was performed in triplicate on three different aBL units.

### 2.4: Device 4.0

Although Device 3.0 was sufficient for *ex vivo* experiments, it was far too big to be used *in vivo*. Subsequently, Device 4.0 incorporated the LED changes established by Device 3.0, but its cooling system and circuitry were modified to reduce overall size; no larger than 4” x 4” x 4” and less than 0.5 ± 0.1 lb. The *in vivo* animal, sheep, also needed to be able to move around freely so the device was modified to function off battery power. Lastly, the sheep were unlikely to treat the devices gently. Additional features were implemented to prevent frequent breaks and allow for quick fixes–in short, “sheep-proofing” the unit. Animals used in preliminary device testing were prospectively approved by IACUC (number 10166) and ACURO (number USUHSFY21007.e001). Animal work was performed under a separate project. Device 4.0 was tested multiple times on 14 different sheep (1000+ h of cumulative use). Pictures provided herein were collected from the presently unpublished study.

The active cooling system was the bulkiest part of Device 3.0. To minimize system complexity and reduce power consumption (two considerations that were immaterial in the *ex vivo* setting but important for *in vivo* use), the Peltier elements and their associated feedback temperature regulation components were eliminated from Device 4.0. They were replaced by a compact, fan-based cooling system with a customized heat sink. COMSOL simulations were performed on four different fin geometries for the heat sink–no fins, short plate fins, short pin fins, and long plate fins. The COMSOL simulations utilized the following base parameters across all tests: convective heat flux (h) = 10 Wm^2^·K, heat source from LEDs = 3 W, ambient temperature = 293.15 K, material (all components) = aluminum, steady state conditions, and no thermal interface materials. The COMSOL parameters were chosen arbitrarily, as the goal of the simulation was to compare the relative performance of different heat sink geometries–not to determine absolute values.

Without temperature regulation components, the new system was subject to temperature fluctuations depending on room temperature, animal temperature, and length of use. Subsequently, final fin geometry was selected such that it would maintain the base of the LEDs below 37°C regardless of other factors. However, the incorporation of fins increased the device’s overall geometrical complexity and could not be manufactured by traditional means. Instead, a 3D-printable aluminum alloy (AlSi_10_Mg) with thermoconductivity properties similar to pure aluminum was used by Protolabs (Maple Plain, MN) to manufacture Device 4.0. The heat test (Section 2.1) was repeated on Device 4.0 to confirm heat dissipation capabilities of the new cooling system.

**Fig 6:**
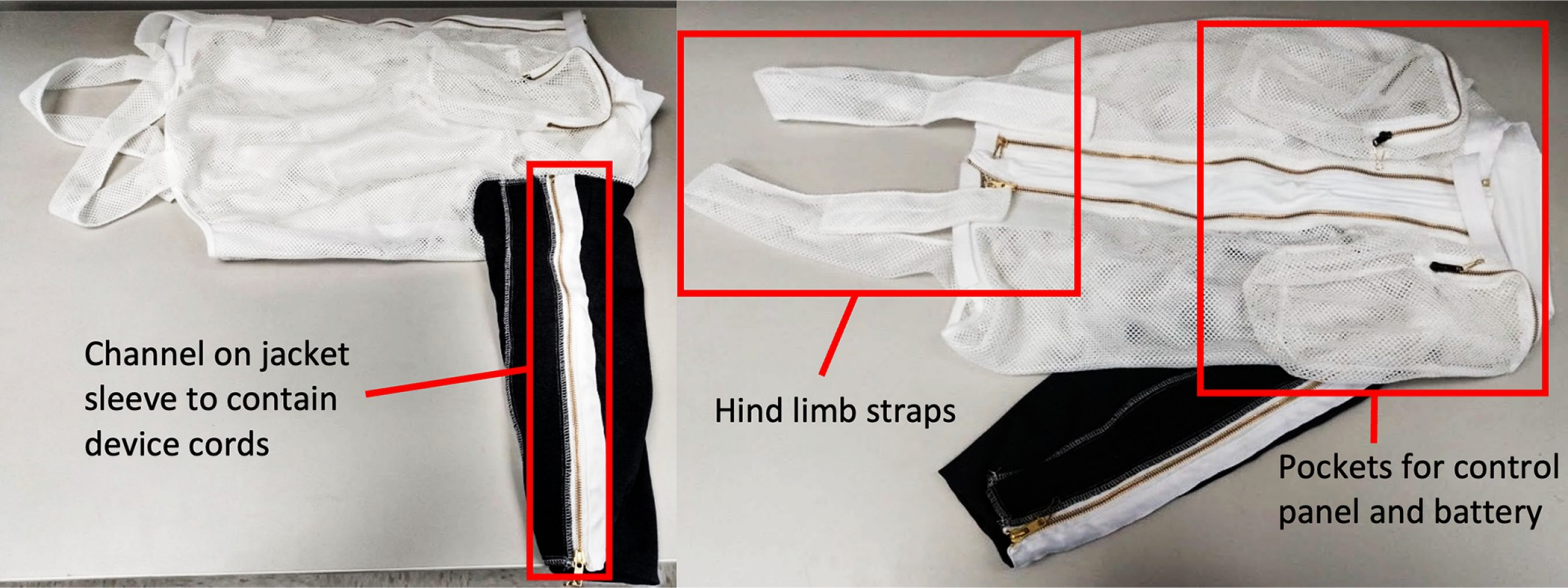
Left, side view and right, top view. Sheep jacket was used to hold the aBL device in place and minimize the sheep’s ability to damage it.

The first step in sheep-proofing the overall system was to design a jacket to hold all device components and allow unrestricted movement (Fig 6). Two shoulder pockets held the battery and LED control panel. A single sleeve slid over the amputated leg and had a zippered pocket to protect the cords connecting the aBL unit on the hoof to the control panel. Lastly, hind straps prevented the jacket from riding up the sheep’s back. The jacket was assembled and sewed by Lomir Biomedical (Notre-Dame-de-l’Île-Perrot, QC).

**Fig 7:**
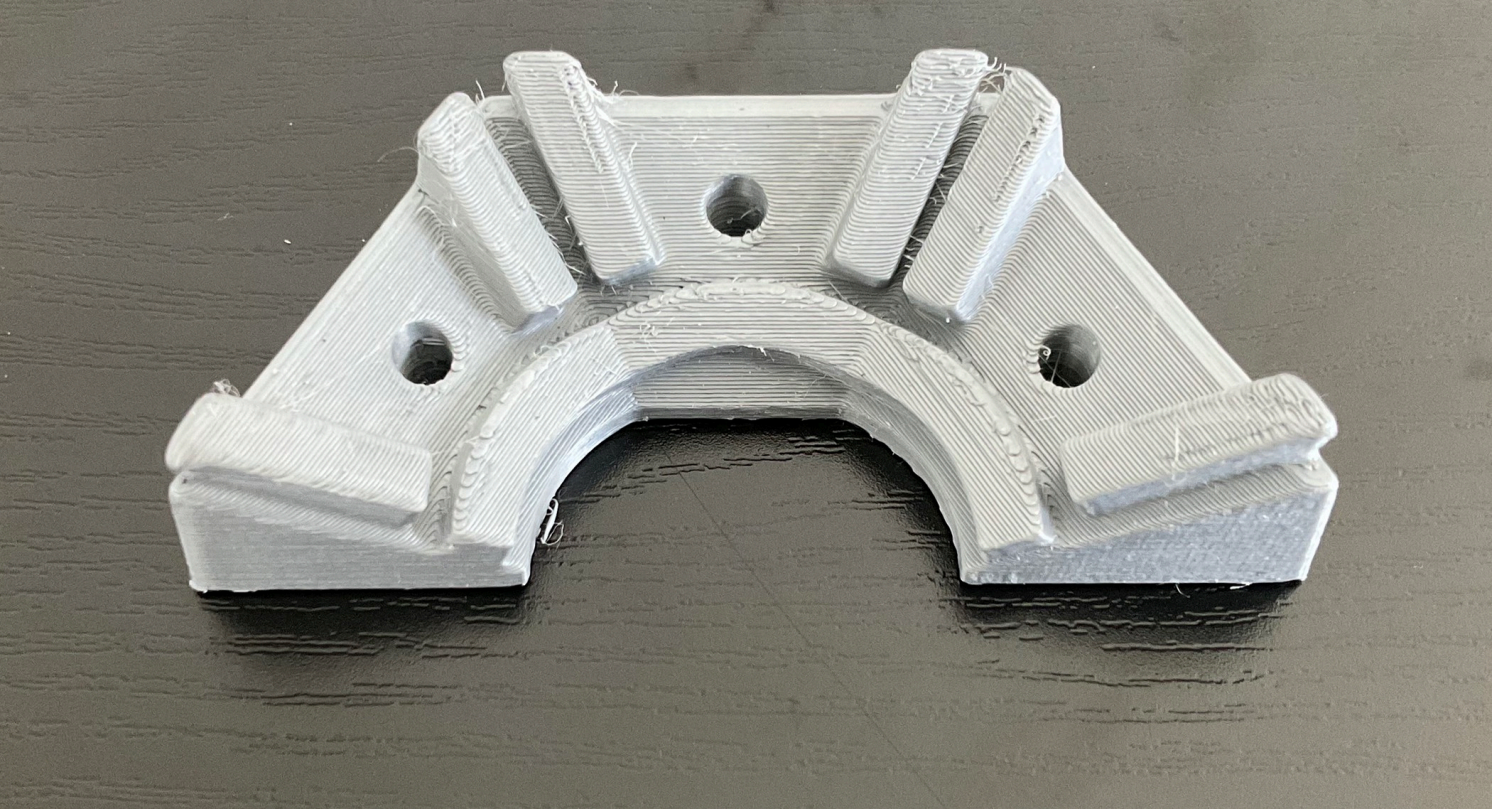
3D printed mold to create thermoformed plastic covers used to cover the LED PCBs.

The constant exposure to water and sheep fluids made it necessary to protect the LED PCBs from frequent electrical shorts. Transparent thermoform plastic covers were designed to cover the LED PCBs and were offset 1–2 mm from the surface. A mold was drafted in Siemens NX (Plano, Texas) and 3D printed in polylactic acid (PLA; see Fig 7).

**Fig 8:**
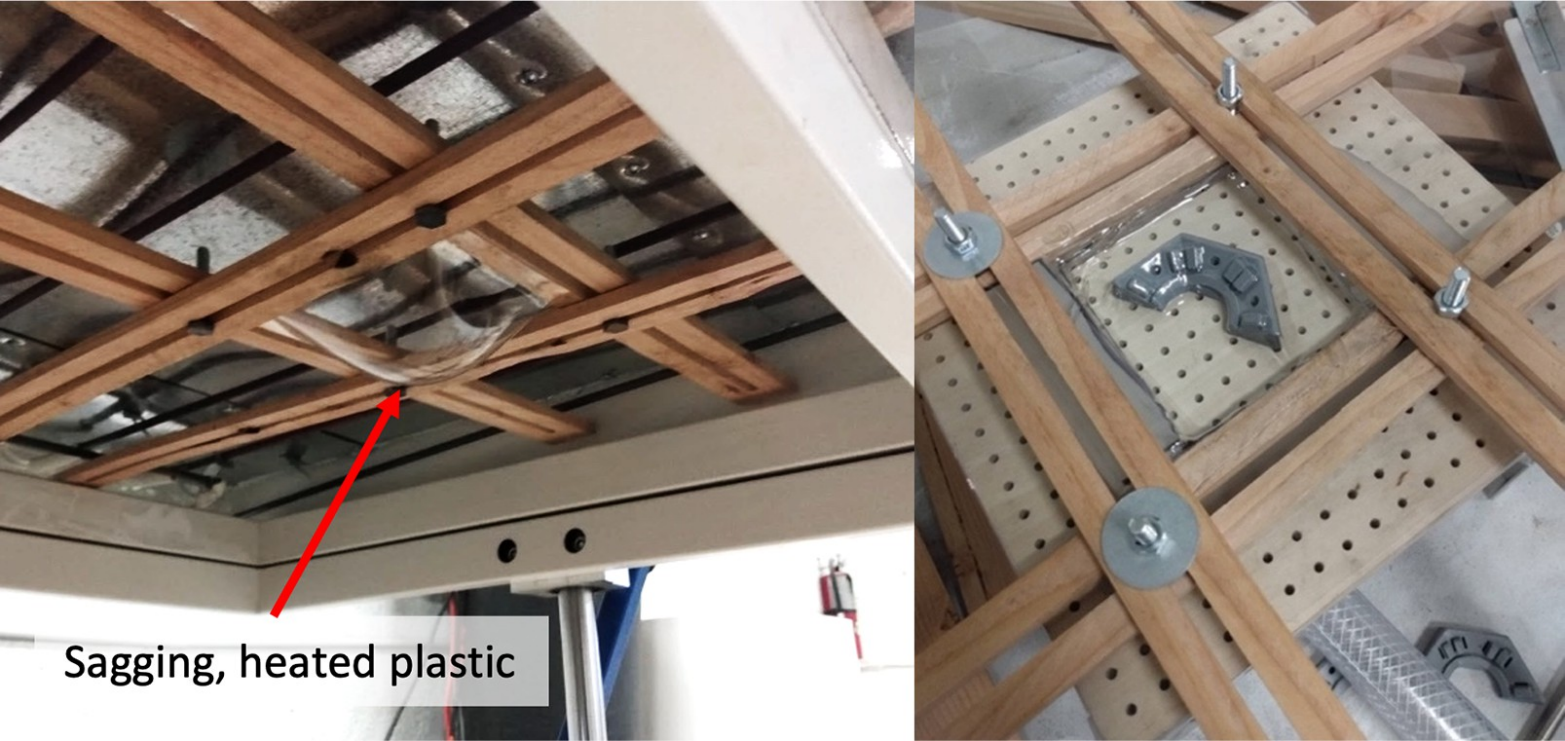
Thermoforming process: Dental plastic was held by a wooden frame and heated under hot coils until the plastic began to sag (left). Plastic was quickly transferred to a vacuum table where the plastic was vacuumed down to fit the shape of the 3D printed mold (right).

**Fig 9:**
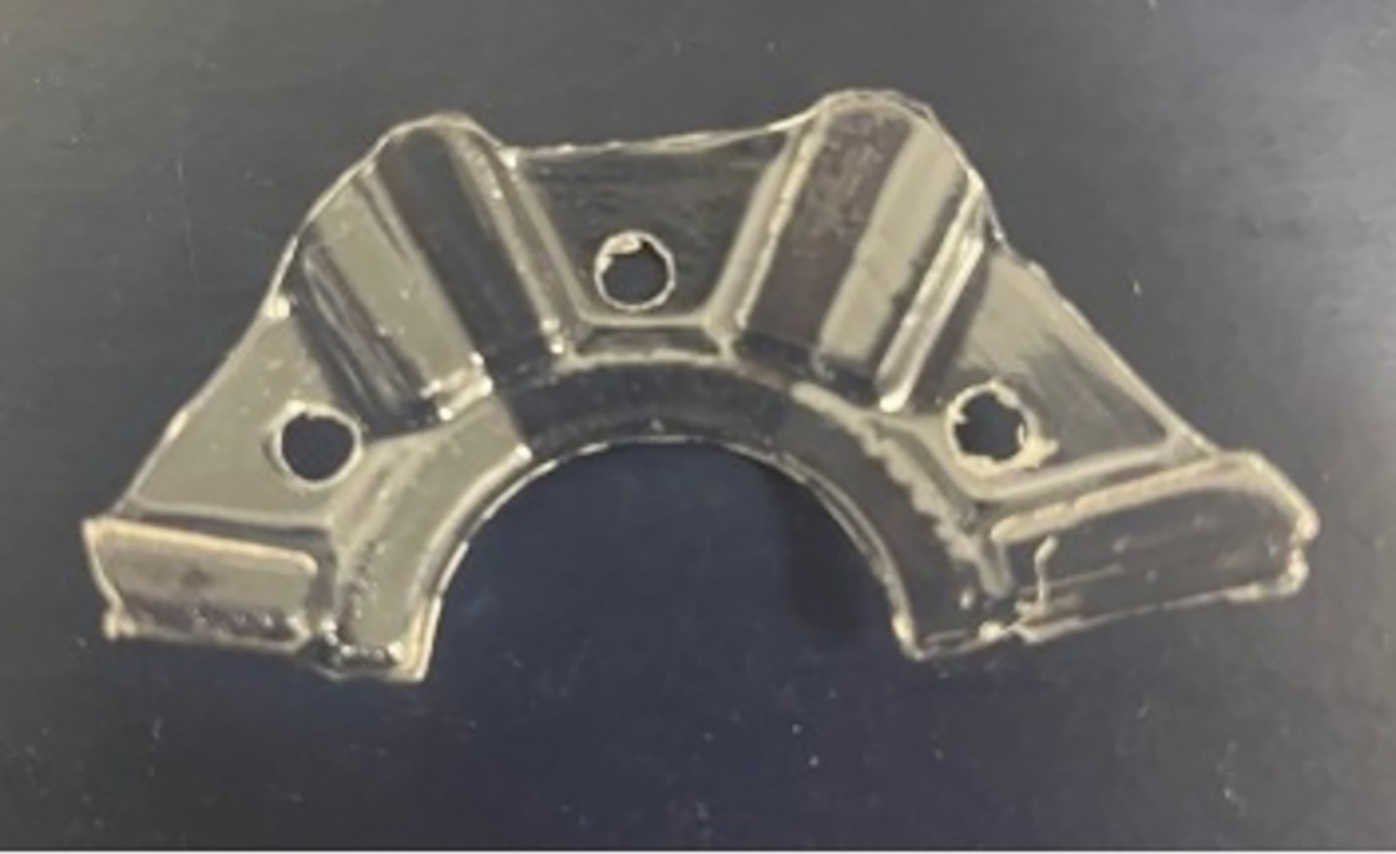
Transparent cover with excess plastic trimmed away and holes drilled through for attachment purposes.

Dental thermoforming plastic (1 mm thick; Impressive Smile, CA) was vacuum formed over the 3D printed mold (see Fig 8). Excess plastic was cut away and holes were drilled out to anchor the LEDs on the aluminum base (see Fig 9). Five individual covers were selected randomly to assess thickness variability between the covers. The LED tilt test (Section 2.2) was also repeated to verify that the addition of the plastic covers did not significantly alter optical power output.

**Fig 10:**
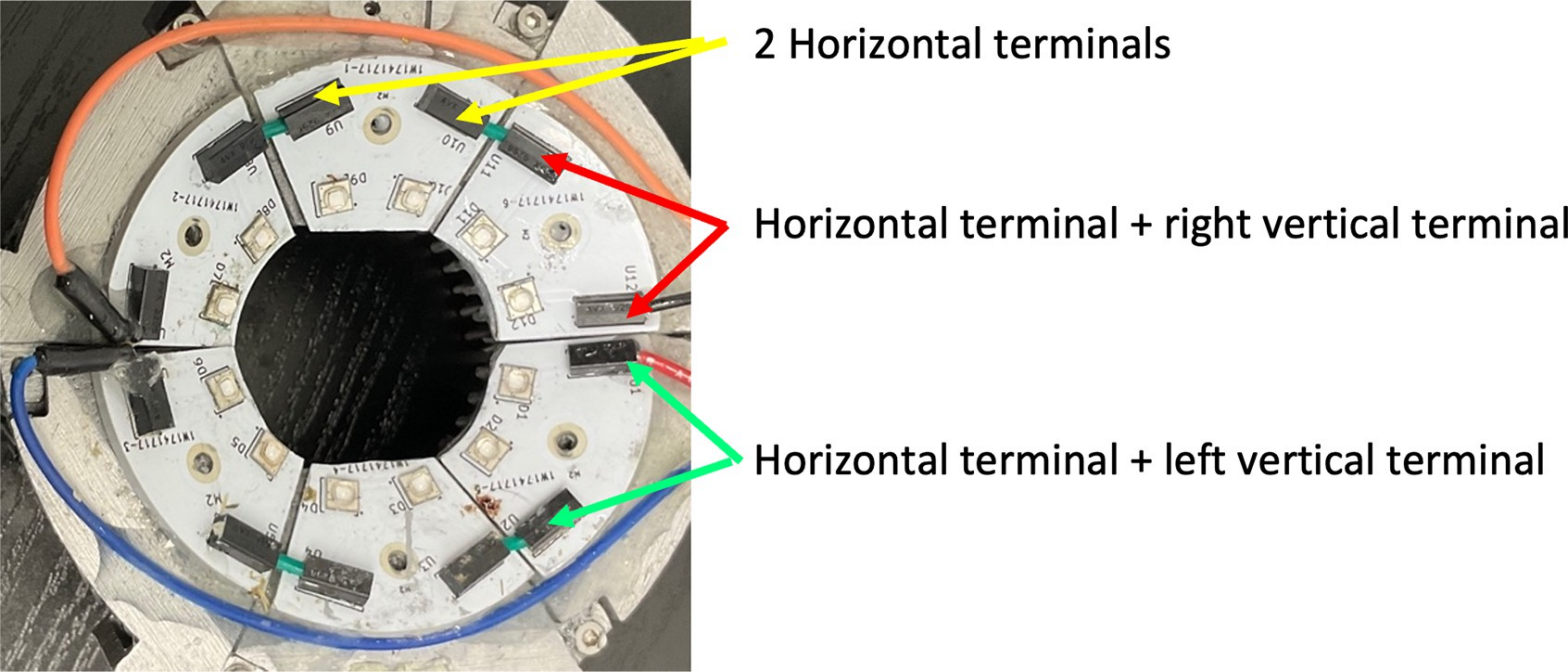
Device 2.1 LEDs, using PCBs with three different wire terminal orientations (black rectangles). This limited the ease of PCB replaceability.

**Fig 11:**
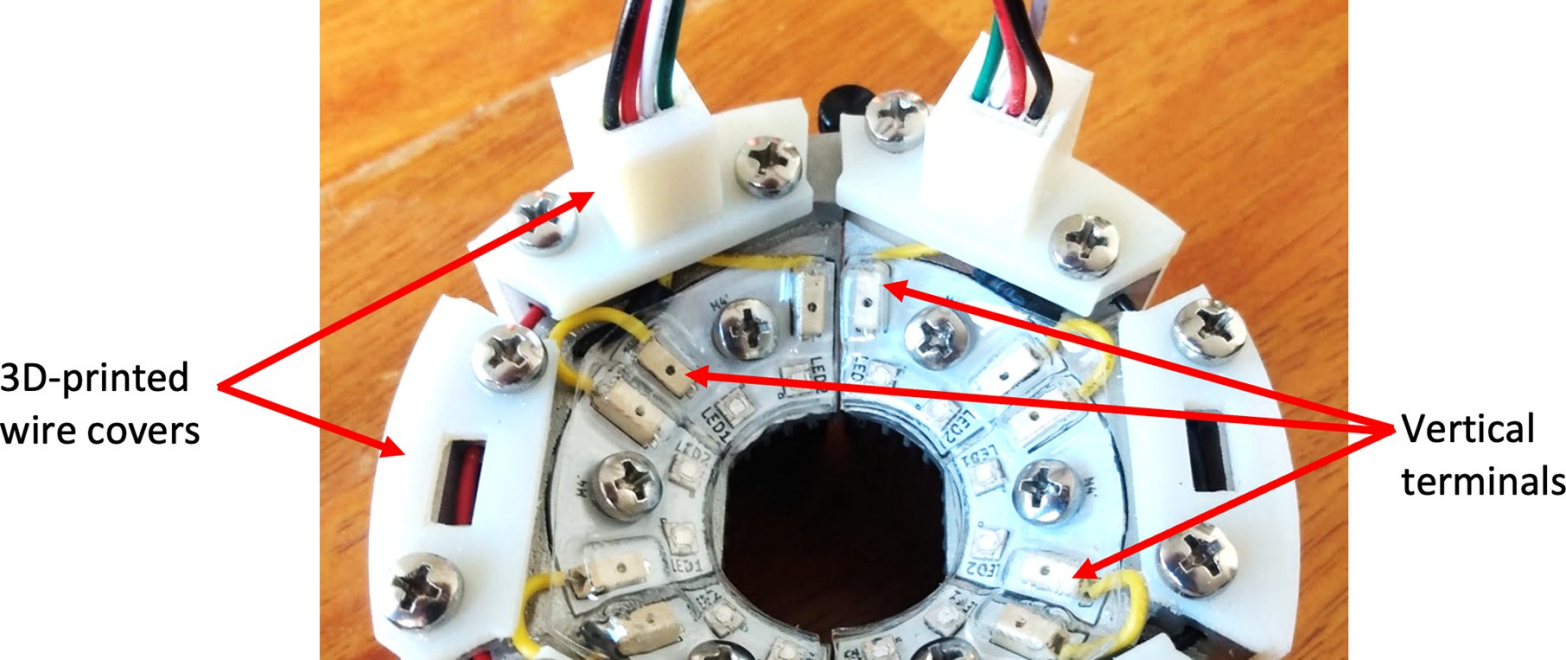
Device with 3D-printed wire covers and LED PCBs with vertically positioned wire terminals. A single LED PCB could be inserted into any of the six possible positions.

While the plastic covers decreased the rate of LED PCB replacement, pilot test sheep inevitably damaged the LEDs. PCB designs with three different wire terminal orientations were used initially (Fig 10) but keeping a steady supply of the three unique LED PCBs added unnecessary complexity to device upkeep. All wire terminals were thus rotated into a vertical position, which made the device more modular as one PCB could be used for any section on the device (see Fig 11).

**Fig 12:**
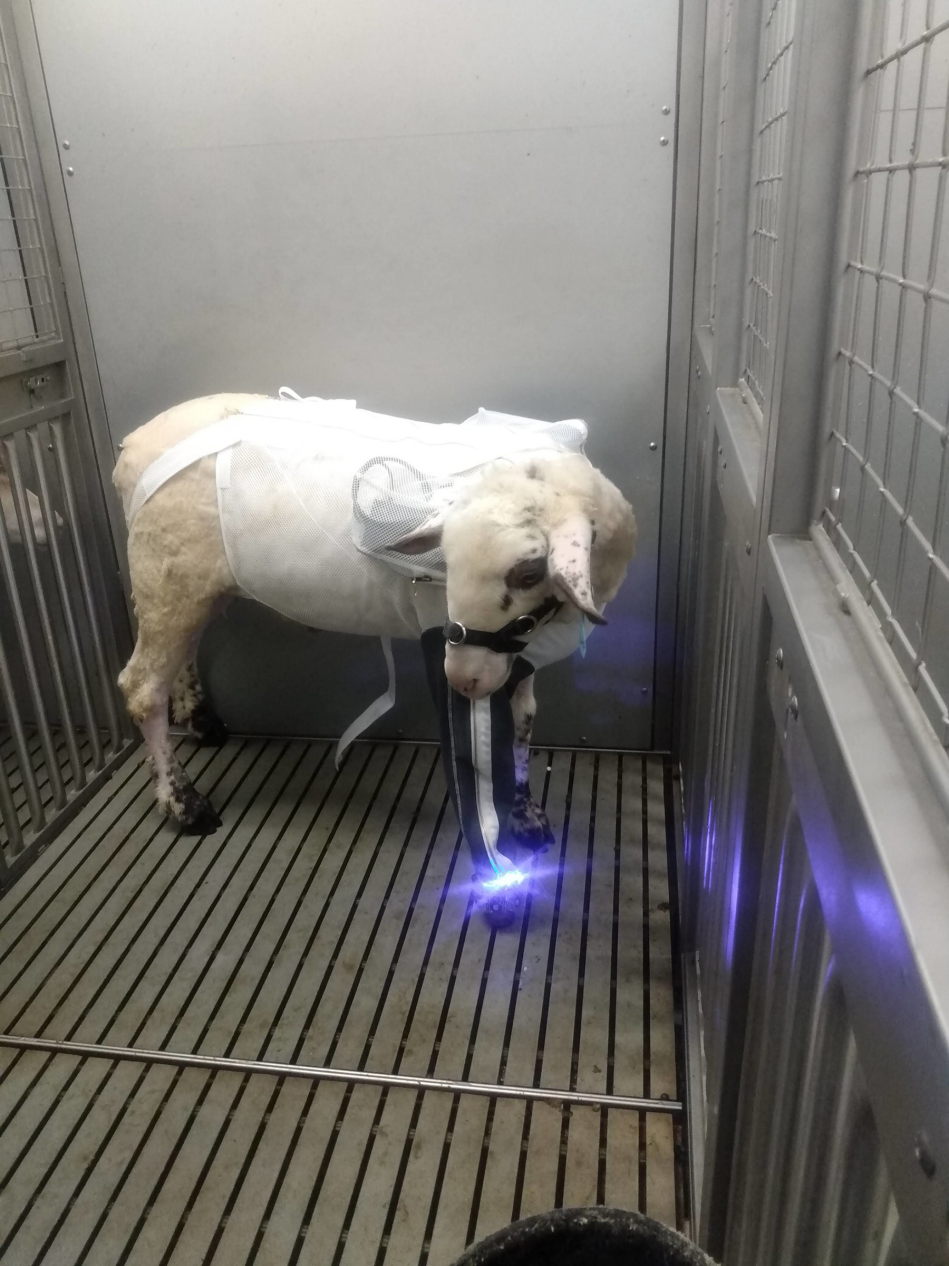
Device 4.0 that was placed on a study animal using the sheep jacket. The aBL device attached around the prosthetic hoof and the cords were threaded up through the zippered pocket on the leg sleeve. The cords were connected to a control panel located in the right-side pocket (pictured). Another cord then connected the device to a 48 V (1000 mAh) battery inserted into a pocket on the left shoulder.

Lastly, improvements were made to prevent wire snapping from aggressive sheep movement. Stereolithography (SLA) 3D printed covers encased the wires around the device and strengthened weak points (see Fig 11). Multiple connection points were added along the wiring system so that in case a wire was forcefully tugged, it would disconnect instead of breaking. The assembled device was then placed on a sheep as seen in Fig 12.

## 3 Results

### 3.1: Device 1.0

The geometry of Device 1.0’s aluminum base allowed the unit to fit snugly around the adapter screw without moving. The LEDs were spaced appropriately so that no noticeable gaps or fluctuations in brightness were observed.

**Fig 13:**
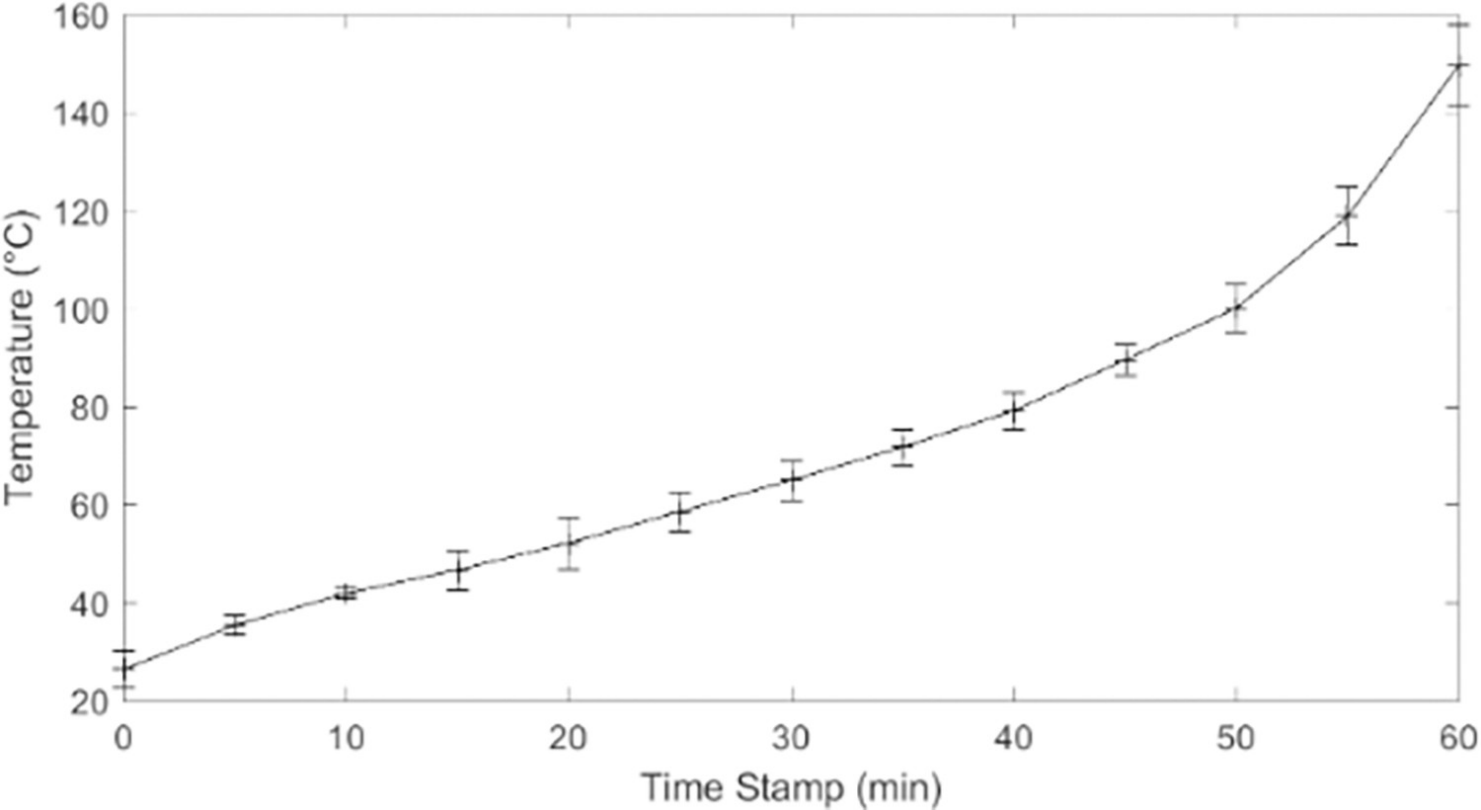
Results of Device 1.0 heat test.

LEDs convert most of their electrical energy consumption to heat and a significantly smaller percentage to light. Subsequently, using 12 LEDs close together to produce 200 mW of optical power output meant that significant electrical energy was simultaneously being converted to heat, for which the aluminum base was ill-equipped to dissipate. The heat test showed that Device 1.0 exceeded body temperature (37°C) within 15 min and 150°C by 1 h (Fig 13). These intense temperatures could result in 3^rd^ degree burns even if patients do not directly touch the aluminum. Additionally, initial *ex vivo* experiments revealed that excess heat contributed to skin desiccation. This heat-killed/pasteurized the inoculated bacteria independent of the aBL treatment (pasteurization occurs at 60°C and above [13]). These two points were the rationale for adding an active cooling system to Device 2.0.

### 3.2: Device 2.0

*Ex vivo* microbiology experiments using Device 2.0 revealed that while the Peltier modules were carefully chosen according to desk calculations, they were still prone to thermal runaway when input current exceeded 250 mA. Current was thus kept constant at 250 mA for all further *ex vivo* and *in vivo* experiments. This correlated to 150–200 mW of optical power output when measured 1 cm directly above the LEDs. At this level, irradiation levels in published literature could be achieved with 15–30 min of aBL exposure [5–7]. Overall, the active cooling system kept the device consistently cool and minimal skin desiccation was observed.

### 3.3: Device 3.0

**Fig 14:**
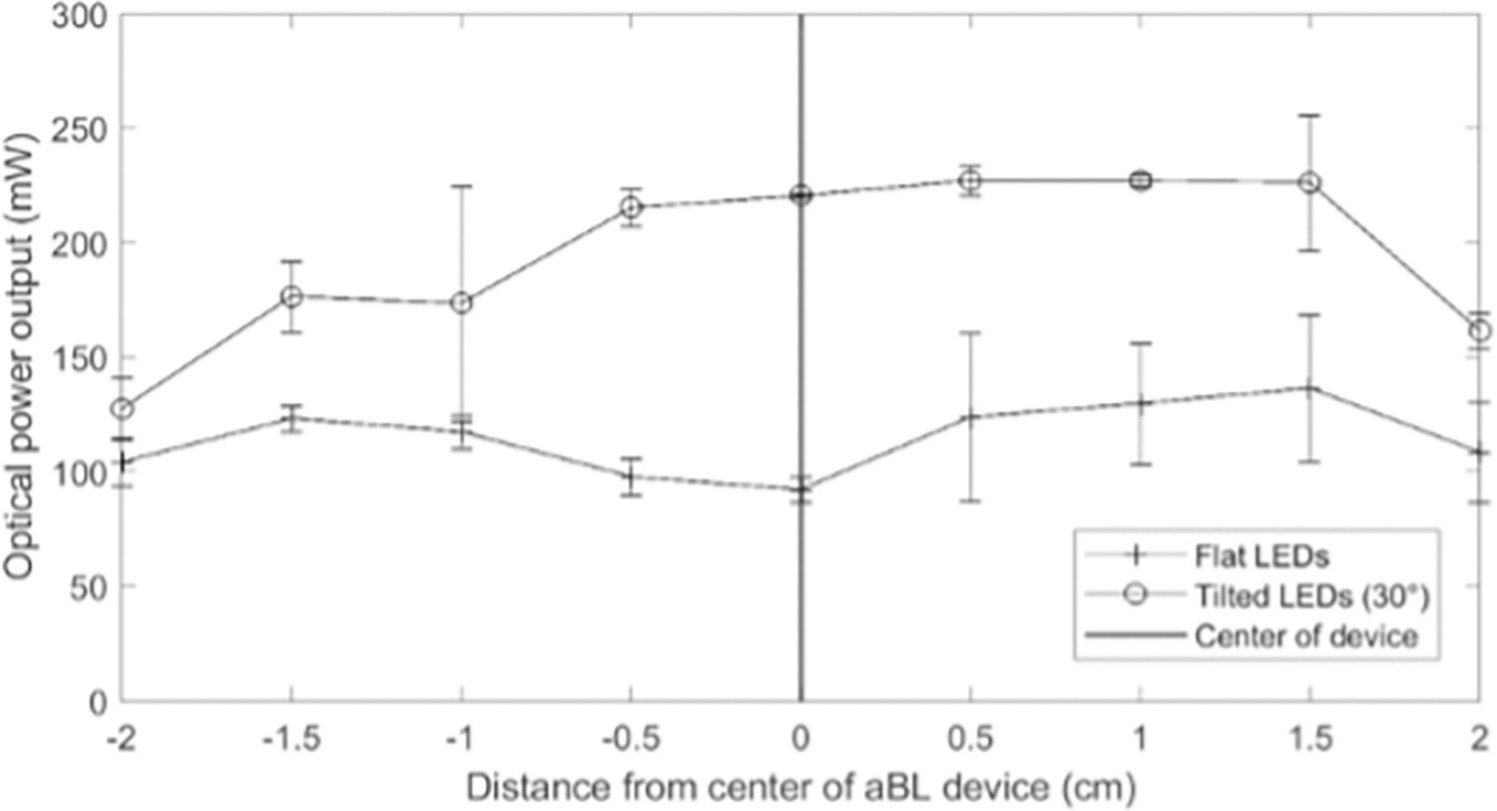
Optical power output was recorded at 0.5 cm increments across the diameter of the Device 2.0 and Device 2.1 –flat and tilted geometries respectively.

The hypothesis that the highest LED optical power output occurred directly above the LEDs was supported by the tilt test (Fig 14). The results indicated that for Device 2.0 (flat LEDs), a dip in optical power output was observed between -1.5 to 1.5 cm but was highest right above the LEDs (around ± 1–1.5 cm). The skin-implant interface was located at ± 0.5 cm, indicating that the flat LED array was underutilizing the LED capabilities and generating wasted optical power output. In contrast, Device 2.1 (LEDs tilted at a 30° angle) had a bump in optical power output between ± 0.5 cm (Fig 14), ensuring that minimal energy was wasted. It was also noted that for both devices, optical power output was higher between 0 to 1.5 cm than between 0 to -1.5 cm, which was unusual due to the symmetry of the aBL device. Potential reasons for this outcome are discussed in Section 4. Overall, observations indicated that the tilt was advantageous, and the device was permanently modified to angle the LED PCBs 30° towards the center of the device (Fig 5).

### 3.4: Device 4.0

**Fig 15:**
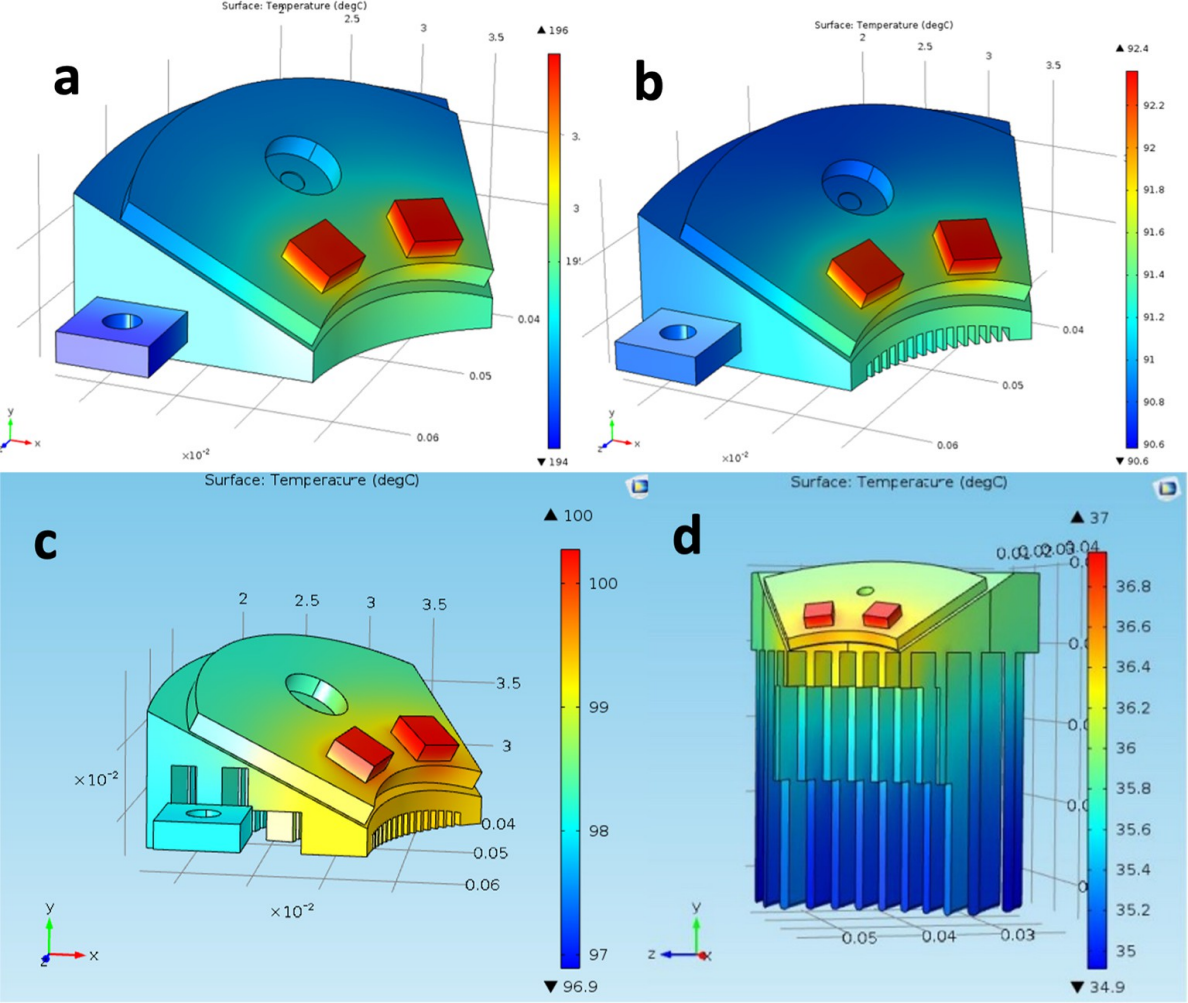
a) No fins; 196°C LED surface temperature, b) Short plate fins; 92.4°C LED surface temperature, c) Pin fins; 100°C LED surface temperature, and d) Three cm long plate fins; 37°C LED surface temperature.

The COMSOL simulations suggested that long plate fins (3 cm) would perform best out of the four geometries tested (Fig 15). Without any fins, very little heat dissipation occurred, and the LEDs were projected to reach 196°C (Fig 15A). Short plate fins reduced the LED temperature to 92°C, which was still far off from the goal of 37°C (Fig 15B). Pin fins, which had significant surface area available, performed worse than the plate fins, only decreasing the LED temperature to 100°C (Fig 15C). Three cm long plate fins (Fig 15D) provided sufficient heat dissipation and attained the LED surface temperature goal of 37°C.

**Fig 16:**
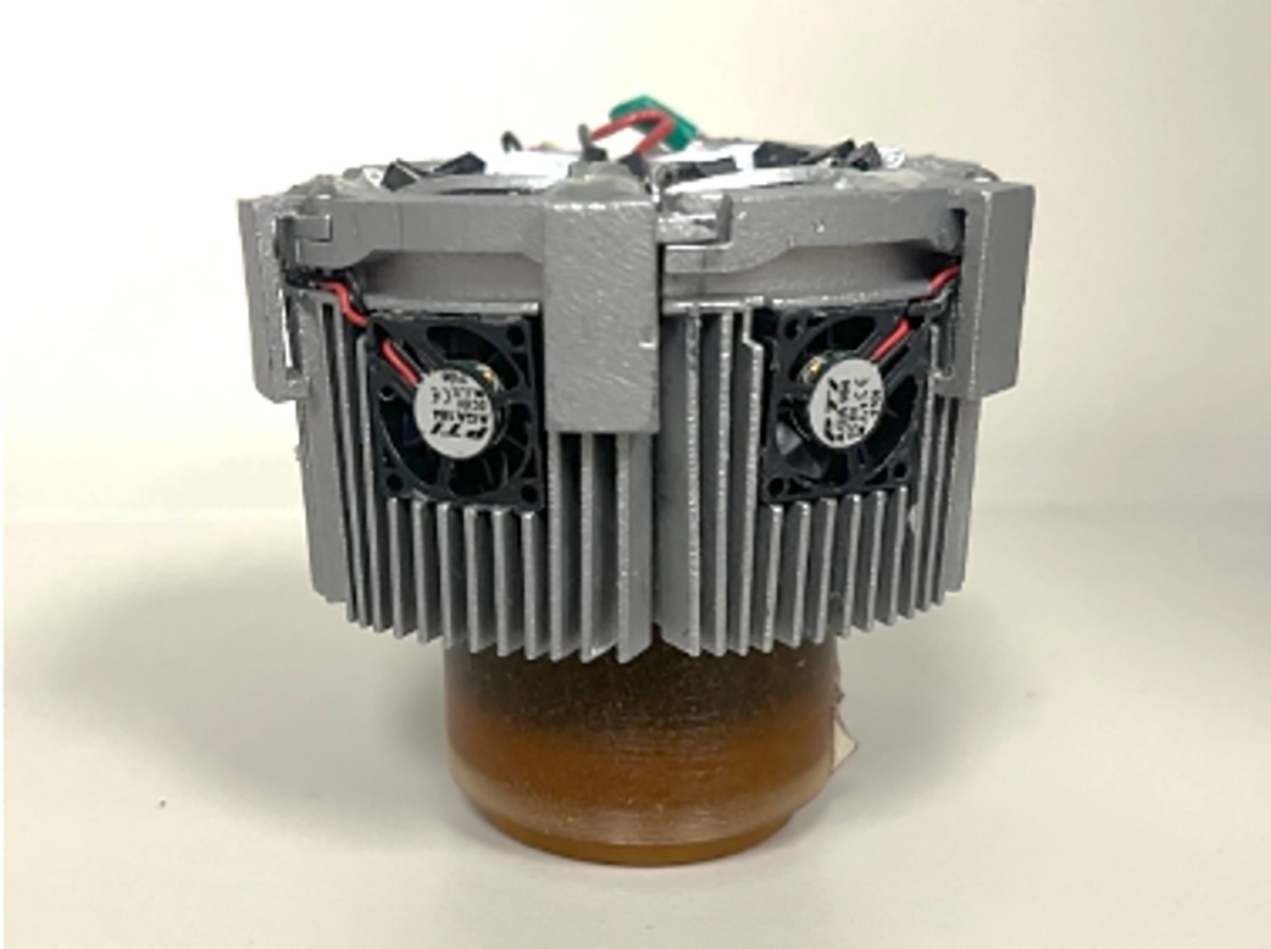
Device 4.0 with 3 cm long plate fins and small fans.

**Fig 17:**
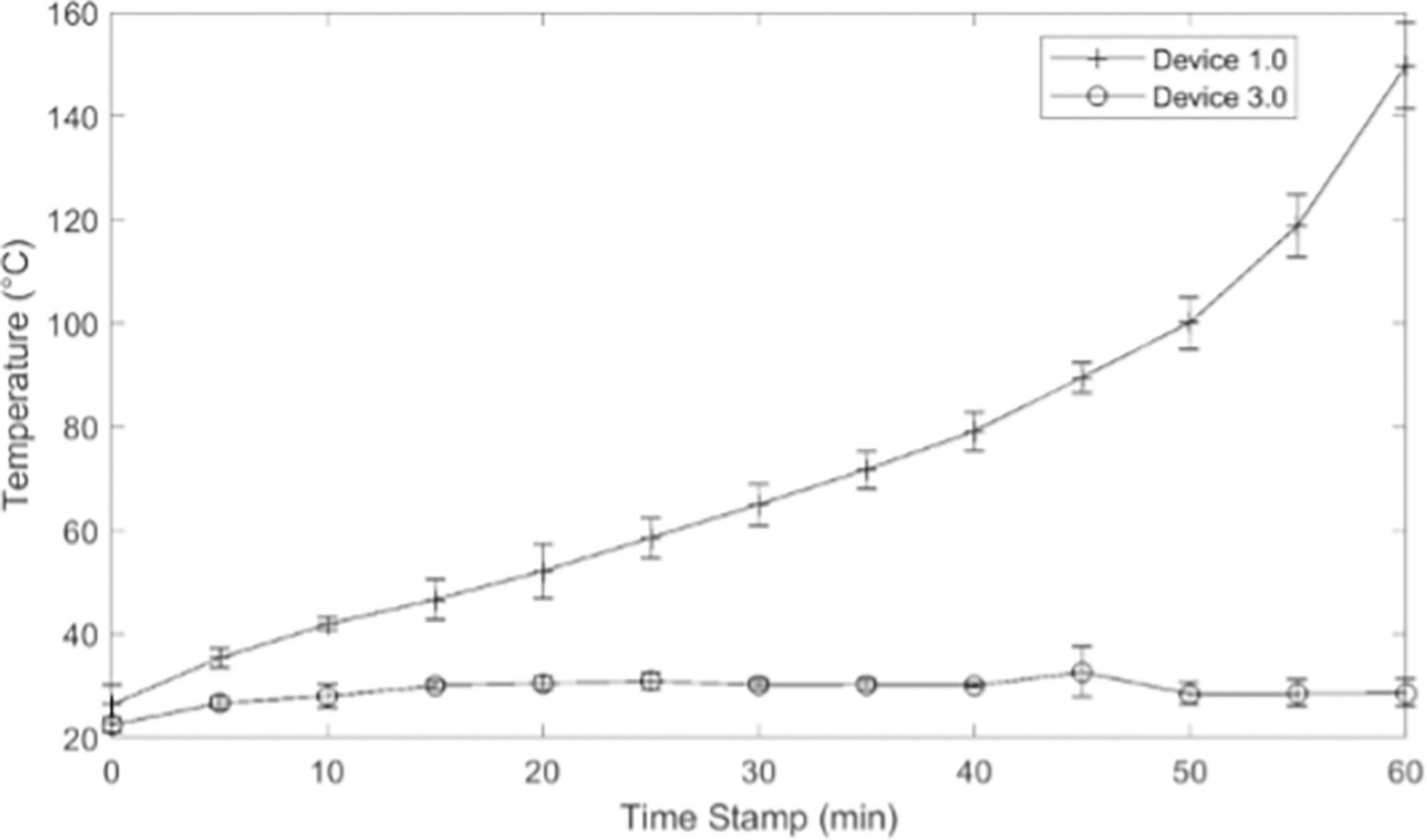
Device 4.0 heat test results overlaid on results from Device 1.0.

Coupled with the 3 cm long plate fins were small fans which increased the overall chilling abilities of the cooling system (Fig 16). When printed out of the aluminum alloy, Device 4.0 weighed 0.66 lb, which was slightly over the specified weight goals, but within the dimensional requirements. When the heat test was performed on Device 4.0 (without small fans), 5–8°C temperature increases were observed within the first five minutes, after which the long plate fins maintained the temperature at approximately 30°C. This validated the COMSOL simulation results and the thermal conductivity properties of the aluminum alloy (Fig 17). It met the 37°C goal and was a 120°C improvement over Device 1.0. Hours of testing this new cooling system *in vivo* showed that the devices were occasionally lukewarm to the touch, yet the sheep skin never exceeded 37°C (measured with a thermal sensor).

#### 3.4.1 in vivo testing on Device 4.0

Over multi-day aBL treatments, the increase in modularity between Devices 3.0 and 4.0 was a key component for performing the *in vivo* studies. The device was used on multiple sheep for 14-day treatments. Each day, aBL was administered for 6 h, with minimal interruptions (quick LED or wire replacement for ones that would break mid-treatment). Device 4.0 allowed the collection of a unique data set that assessed the potential of aBL to manage biofilm burden at the skin-implant interface of percutaneous OI implants.

LED replacement was quick and easy with the vertically oriented wire terminals, enabling PCB replacement within minutes. Unfortunately, the release mechanisms made to safeguard weak points in the wiring system did not work as anticipated and resulted in longer repair times. Wires would often break directly behind the release mechanisms instead of coming undone. Due to the length of the wires between junctions, it was easier to solder on a new connector instead of replacing the entire wire segment. Altogether, wire repair times delayed treatment anywhere from an hour to a day.

The sheep jacket was tested on multiple sheep with positive results. Cords were sufficiently protected from teeth and no obvious discomfort was observed. A few additions were made to account for variations in sheep size, including hind strap extenders and back panels. Unfortunately, there was no way to shorten/lengthen the sleeve due to the zipper channel. If the sleeve was too short the sheep would occasionally bite through the protected wires, which could be addressed with soldering. The more problematic issue was when the sleeve was too long; the sleeve was prone to sag around the LEDs and trap heat. This compromised the cooling system and made the device too hot to touch. Pinning up the sleeve helped, but the zipper channel could not be properly used which increased chances for bitten wires.

Lastly, all sampled plastic LED covers were similar in thickness and measured 0.037 ± 0.0065 in. A repeat of the LED tilt test with plastic covers showed decreases in optical power output up to 50 mW. Nevertheless, it should be noted that the tested covers had already been in use for at least one week. The covers were cleaned daily with a sanitary wipe, but certain scratches and sheep fluids were immovable. Covers were replaced approximately every week, indicating that power output reductions likely maxed out around ~50 mW.

## 4 Discussion

Percutaneous OI implants are highly susceptible to frequent infections since they breach the skin barrier and provide a channel through which bacteria in the environment may easily access the inner body. Dealing with constant irritation, discomfort, and pain associated with infection, an patient’s quality of life is greatly hindered [14]. Subsequently, aBL has been suggested as a way to manage bacterial burden at the skin-implant interface and decrease the likelihood of infection. However, this antimicrobial option has not yet been investigated as no available aBL device is equipped to target the skin-implant interface of percutaneous OI implants. Structural aspects of available aBL systems are non-translatable for efficient treatment in individuals with OI implants and the systems lack the optical power necessary to kill biofilms often present at the skin-implant interfaces. In response, we developed an aBL device that overcame the structural and optical limitations of current aBL systems.

The structural design established by Device 1.0, which connected the unit’s two halves together around the prosthetic adapter screw, was a straightforward process achieved without undocking the prosthetic. Adapted by Device 4.0, this strategy helped the unit fit snugly in place while sheep moved freely about their pen as their skin-implant site was evenly irradiated. However, the device halves were held together with small screws. While they effectively connected the halves, the screws were easily dropped while putting the aBL device on the sheep. If this aBL unit is to be used in human clinical trials, the screws are small enough, and the device placed at a sufficiently distal position that a patient is likely to find the attachment process troublesome. Implementing latches or snaps for future device iterations would be optimal.

The angled LEDs used in Device 3.0 helped with both structural and optical aspects of the aBL device. The ring of 12 LEDs provided even irradiation while the 30° angle ensured that sufficient optical power was directed towards the skin-implant site. Nevertheless, validating the hypothesis that maximum optical power output occurred directly above the LEDs to provide rationalization for the 30° angle, was challenged by the slight right-skew of the tilt-test data (Fig 13); measurements were higher between 0 to 1.5 cm than 0 to -1.5 cm on both flat and angled devices. At face value, this implied that the LEDs did not provide even irradiation around the entire ring. However, closer examination revealed that the skewed data was more likely due to limitations of the power meter as opposed to imbalance in device irradiation. For each of the three trials, a different unit was tested, each at a unique orientation. Subsequently, a different side of the device was in the 0 to 1.5 cm range for every test. The power meter orientation never changed and may have read wattage better from light entering the right side than from the left. It was thus concluded that it was altogether better for the LED angle to be permanently adjusted to 30°. This made it so biofilms at the skin-implant interface of the OI ovine implant model would be targeted with high aBL fluences while simultaneously minimizing energy waste.

The cooling system implemented in Device 4.0 enabled high optical levels without overheating. However, early experimentation in the OI sheep model revealed a major drawback that needs to be fixed if translated to human clinical trials. If the LEDs were in any way covered by the jacket sleeve, the device became too hot to touch, even if the fins and fans had not been affected. In the animal model, the sleeve could be pinned up and overheating was preventable. However, it would be complicated to ensure that the LEDs are never covered during use by individuals with OI implants. Patients would be restricted to wearing shorts and have to refrain from relaxing with a blanket while receiving aBL treatment. Simple activities such as laying on a couch or in thick grass could also compromise the device. Overall, a patient’s ability to use this device at any time throughout the normal day is limited. Additionally, a person with amputation has a lot more muscle and tissue around the OI implant in comparison to the *in vivo* sheep model. They will therefore have tissue that naturally “sags” around the implant which may trap heat even if all precaution is taken to avoid long pants and blankets while receiving aBL treatment. It is therefore essential to perform an in-depth analysis of the heat dissipation problem before implementing this aBL device in human clinical trials. An increased understanding of the issue may indicate only minor modifications are necessary, although complete restructuring of the entire device could be equally possible.

Device size, wire terminals/connections, and the plastic covers were not necessary features to meet the three design goals outlined in Section 1.0 but were still essential parameters to the overall functionality of the device in an OI sheep model. The downsized cooling system from Device 3.0 to Device 4.0 allowed the aBL unit to meet the desired dimensions (4”x4”x4”), although the length of the fins caused the system to go slightly above the weight limits. Nevertheless, this did not cause any adverse effects such as increased bleeding at the implant site or premature undocking of the prosthetic hoof. The smaller geometry of Device 4.0 was thus adequate to carry out *in vivo* aBL experiments, but animal responses made it clear the device was still too heavy/large. Irritated by the Device 4.0’s present bulk, sheep would constantly stomp and whack their legs in an attempt to knock it off, increasing the rate of part breakage. If the sheep laid down, their legs rested at awkward angles as the device was noticeably thicker than their legs. Overall, further downsizing efforts to make the weight less noticeable and improve comfort in additional sheep and human experiments is another aspect that should be noted.

LED PCBs with vertical wire terminals enabled straightforward replacement but a person had to have steady hands and special tools. For an animal study, this methodology was acceptable, but for individuals with amputation, it is problematic. A replacement method similar to putting in a new battery would be better in future prototypes. Furthermore, the soldering required to fix broken wires was an issue that also needs to be improved. The present connection points were enough to prevent major delays in treatment (> 1 day), but aBL therapy was interrupted for at least 1–2 hours if a wire broke mid-treatment. Device 4.0 may therefore benefit from incorporating shorter wire segments so that entire wire segments can be replaced instead of soldering on new connection points. Additionally, the wiring system could be re-structured to place all the wires in a single thick sheath instead of being separated into thinner, easily breakable sections.

Lastly, the plastic covers were vital to maintaining uninterrupted aBL treatment, but their decrease in optical power output (~50 mW) was concerning. With Device 4.0, the 50 mW optical power output reduction was inevitable if daily LED PCBs replacement was to be avoided. While new covers could be swapped in every morning before use, the holding screws were tedious to undo, and significant scratches from everyday sheep movement were evident within an hour of use. More data is necessary to optimize the design; it is possible the 50 mW decrease in optical power output (which is the higher end) may not significantly affect aBL treatment. Alternatively, if the difference is significant, treatment time could be adjusted to attain the desired irradiation parameters (J/cm^2^) at a lower optical power output. Nevertheless, alternative materials for the covers that are more scratch resistant should be explored as adjusting irradiation parameters to compensate for lower optical power will extend the time a patient must wear the aBL device. Importantly, these considerations may be irrelevant in the human application as a cover may not be needed.

Altogether, Device 4.0 provided the necessary technology to perform *in vivo* aBL experiments for infection management at the skin-implant interface of percutaneous OI implants (manuscript containing the microbiology data is in-draft). aBL treatment, using a clinically relevant treatment period (14 days), was compared to other common antimicrobial treatments such as antibiotics or daily soap and water washing. Device 4.0 thus established an important step in aBL research for eventual human application and may also be easily modified as a method of studying other light therapies in percutaneous applications. For example, LEDs of different wavelengths could be used to optimize photodynamic therapy. Alternatively, the device could be tweaked to fit around OI implants in other animals or humans, or even used for other percutaneous device applications like feeding tube ports. Altogether, the device outlined in this manuscript may be used to significantly expand antimicrobial light therapy research.

### 4.1: The future of Device 4.0

Although Device 4.0 was used for 6 h applications in the ovine model, the parameters for humans has yet to be determined (reasons for this will be published in the forthcoming *in vivo* manuscript). However, it is likely that 6 consecutive hours of device use may be too long for human patients to feel unhindered while being treated. Further alterations to the device to maintain efficacy with shorter treatment times may be necessary (e.g. pulsing at higher irradiations, dual wavelengths [15, 16]).

Additionally, the device is presently shaped to fit around the adapter screw of the ovine OI implant model. When translated to human use, it is hoped that the device would also attach to a connection point similar to the adapter screw. The geometry at this interface, may however, change according to the OI implant system used, or the prosthetic manufacturer. The device can be modified to make room for removable adapters so the light could be used by any patient with an OI implant.

Once necessary adjustments are made to Device 4.0 regarding irradiation time and geometry, the device’s ease of use must also be examined. Ideally, the aBL device would be controlled by the individuals themselves. As such, better contained cords, a decrease in bulkiness, and additional durability factors are important components to be considered. For example, individual wires should not be visible and would decrease damage by environmental factors. The 48 V battery was quite heavy and would be inconvenient for an individual with limb loss to transport during regular daily activities. Other power alternatives may be preferred, such as solar panels, or a smaller, but more expensive lithium battery. Lastly, the rate of plastic cover replacement in the animal model was doable, but the covers should be replaced with a non-scratchable cover that lasts the lifetime of the device. On the other hand, many of the device adaptations that were implemented to protect against the animals biting, kicking, or otherwise treating the device violently, will likely be unnecessary in devices made for humans with limb loss. It would thus be possible to decrease some of the complexity associated with the current device design.

Overall, it is proposed that the aBL device is used for prevention of infection. Once an infection is established, the bacterial load will most likely have penetrated deep within the tissue–beyond the limits of aBL wavelengths. If, however, aBL can decrease bacterial load from daily contamination before the bacteria can travel deep within the skin, it has the potential to mitigate the occurrence of infections surrounding percutaneous OI implants. The *in vivo* microbiology work performed using this aBL device expounds on aBL’s antimicrobial efficacy and will be submitted for publication later this year.

## 5 Conclusion

aBL is an attractive option to manage biofilm-related infections surrounding percutaneous OI implants, but individuals with OI implants cannot use marketed aBL devices due to limitations in structure and optical power. This paper details a unique aBL device concept that overcame structural and optical limitations of current systems by incorporating design features that could evenly irradiate the entire circumferential skin-implant interface of OI implants and have enough optical power to kill biofilms. The completion of *in vivo* animal data utilizing Device 4.0 will help ascertain the potential of aBL to manage biofilm-related percutaneous OI implant infections. Depending on the success of *in vivo* research using this device, the system will be further improved for human clinical trials. This will help progress infection prevention research for percutaneous OI implants and provide a potential option for improving the quality of life for individuals with OI.
